# Diagnostic Value of MRI Features in Dual-phenotype Hepatocellular Carcinoma: A Preliminary Study

**DOI:** 10.1007/s10278-023-00888-9

**Published:** 2023-08-14

**Authors:** Hong-Xian Gu, Xiao-Shan Huang, Jian-Xia Xu, Ping Zhu, Jian-Feng Xu, Shu-Feng Fan

**Affiliations:** 1 Radiology Department, Second Affiliated Hospital of Zhejiang Chinese Medical University, Hangzhou, 310005 China; 2 Department of Radiology, the Peopleʼs Hospital of Jianyang City, Chengdu, 641499 China; 3 Department of Radiology, Shulan (Hangzhou) Hospital, Hangzhou, 310000 China

**Keywords:** Cancer, Hepatocellular carcinoma, Dual-phenotype Hepatocellular carcinoma, Intrahepatic cholangiocarcinoma, Magnetic resonance imaging

## Abstract

This study aimed to explore the magnetic resonance imaging (MRI) features of dual-phenotype hepatocellular carcinoma (DPHCC) and their diagnostic value.The data of 208 patients with primary liver cancer were retrospectively analysed between January 2016 and June 2021. Based on the pathological diagnostic criteria, 27 patients were classified into the DPHCC group, 113 patients into the noncholangiocyte-phenotype hepatocellular carcinoma (NCPHCC) group, and 68 patients with intrahepatic cholangiocarcinoma (ICC) were classified into the ICC group. Two abdominal radiologists reviewed the preoperative MRI features by a double-blind method. The MRI features and key laboratory and clinical indicators were compared between the groups. The potentially valuable MRI features and key laboratory and clinical characteristics for predicting DPHCC were identified by univariate and multivariate analyses, and the odds ratios (ORs) were recorded. In multivariate analysis, tumour without capsule (P = 0.046, OR = 9.777), dynamic persistent enhancement (P = 0.006, OR = 46.941), and targetoid appearance on diffusion-weighted imaging (DWI) (P = 0.021, OR = 30.566) were independently significant factors in the detection of DPHCC compared to NCPHCC. Serum alpha-fetoprotein (AFP) > 20 µg/L (P = 0.036, OR = 67.097) and prevalence of hepatitis B virus (HBV) infection (P = 0.020, OR = 153.633) were independent significant factors in predicting DPHCC compared to ICC. The differences in other tumour marker levels and imaging features between the groups were not significant. In MR enhanced and diffusion imaging, tumour without capsule, persistent enhancement and DWI targetoid findings, combined with AFP > 20 µg/L and HBV infection-positive laboratory results, can help to diagnose DPHCC and differentiate it from NCPHCC and ICC. These results suggest that clinical, laboratory and MRI features should be integrated to construct an AI diagnostic model for DPHCC.

## Introduction

There are three main types of primary liver cancer: hepatocellular carcinoma (HCC), intrahepatic cholangiocarcinoma (ICC) and combined hepatocellular-cholangiocarcinoma (CHC). Hepatocellular carcinoma is the most common type, the fifth most common malignancy worldwide and the third leading cause of cancer-related death [1, 2]. In clinical practice, primary liver cancer has been found to be heterogeneous in terms of genetic and molecular patterns. Some HCC may display typical HCC morphological features but express both hepatocyte and cholangiocyte immunophenotype markers within the same tumour cells. Among the cholangiocyte markers, the expression of CK19 is more common and found in 10–30% of HCC [3–5]. There are a growing number of studies reporting that HCC expressing cholangiocyte markers is more aggressive with a higher rate of recurrence and lymph node metastasis than single-phenotype hepatocellular carcinoma that does not express cholangiocyte markers [6, 7].

HCC with the expression of cholangiocyte markers can be diagnosed and treated as a new separate subtype of HCC, dual-phenotype hepatocellular carcinoma (DPHCC). The pathological diagnosis of DPHCC is as follows: (1) Immunohistochemically, in more than 15% of the tumour cells, at least one hepatocyte marker (such as Hep Par 1) shows strong positive expression and mainly a diffuse distribution. (2) In more than 15% of tumour cells, at least one cholangiocyte marker (such as CK19) and at least one hepatocyte marker (such as Hep par 1) are coexpressed. If the tumour tissue contains any independent hepatocellular carcinoma and intrahepatic cholangiocarcinoma components, regardless of whether a transition zone exists between these components or if the tumour cells do not simultaneously express markers for hepatocellular carcinoma and cholangiocarcinoma, the patients cannot be diagnosed with DPHCC [3–6]. According to the criteria, HCC can be divided into DPHCC and noncholangiocyte-phenotype hepatocellular carcinoma (NCPHCC).

Despite the increasing awareness of the worse prognosis of DPHCC, there are still few studies in the literature reporting the MRI characteristics and accurate assessment of this particular subtype. In the clinical work, we observed some differences in MR dynamic enhancement and diffusion-weighted imaging (DWI) performance between DPHCC and NCPHCC and speculated that these differences may be related to DPHCC cells with double labels of hepatocytes and cholangiocytes, and there may also be some corresponding differences in tumour markers or clinical manifestations or some of the dual biological behavioural properties of HCC and ICC. Combining these differences may help to improve the accuracy of the diagnosis of DPHCC, help in the selection of patients with poor prognosis, and provide the basis for clinical research, the selection of treatment options, and the construction of diagnosis and prediction models based on deep learning.

The purpose of our study was to determine the value of preoperative MRI for the diagnosis of DPHCC and to explore the imaging manifestations and clinical and laboratory features that have significant value in differentiating DPHCC from NCPHCC and ICC.

## Materials and Methods

### Subjects

This retrospective study was approved by the scientific research ethics committee and institutional review board of the hospital, and the requirement for patient informed consent was waived (KY 2021024). All methods were performed in accordance with the relevant guidelines and regulations. We collected 358 patients with pathologically confirmed primary liver cancer from January 2016 to June 2021, and 208 patients were finally included. The inclusion criteria were (1) pathologically proven primary liver cancer (including DPHCC, NCPHCC, ICC) after hepatectomy or liver transplantation in accordance with the Milan criteria, consisting of single and multiple intrahepatic lesions, (2) underwent contrast-enhanced MR examination within one month before surgery, and (3) complete key clinical data, including infectious disease and tumour markers.

**Fig. 1:**
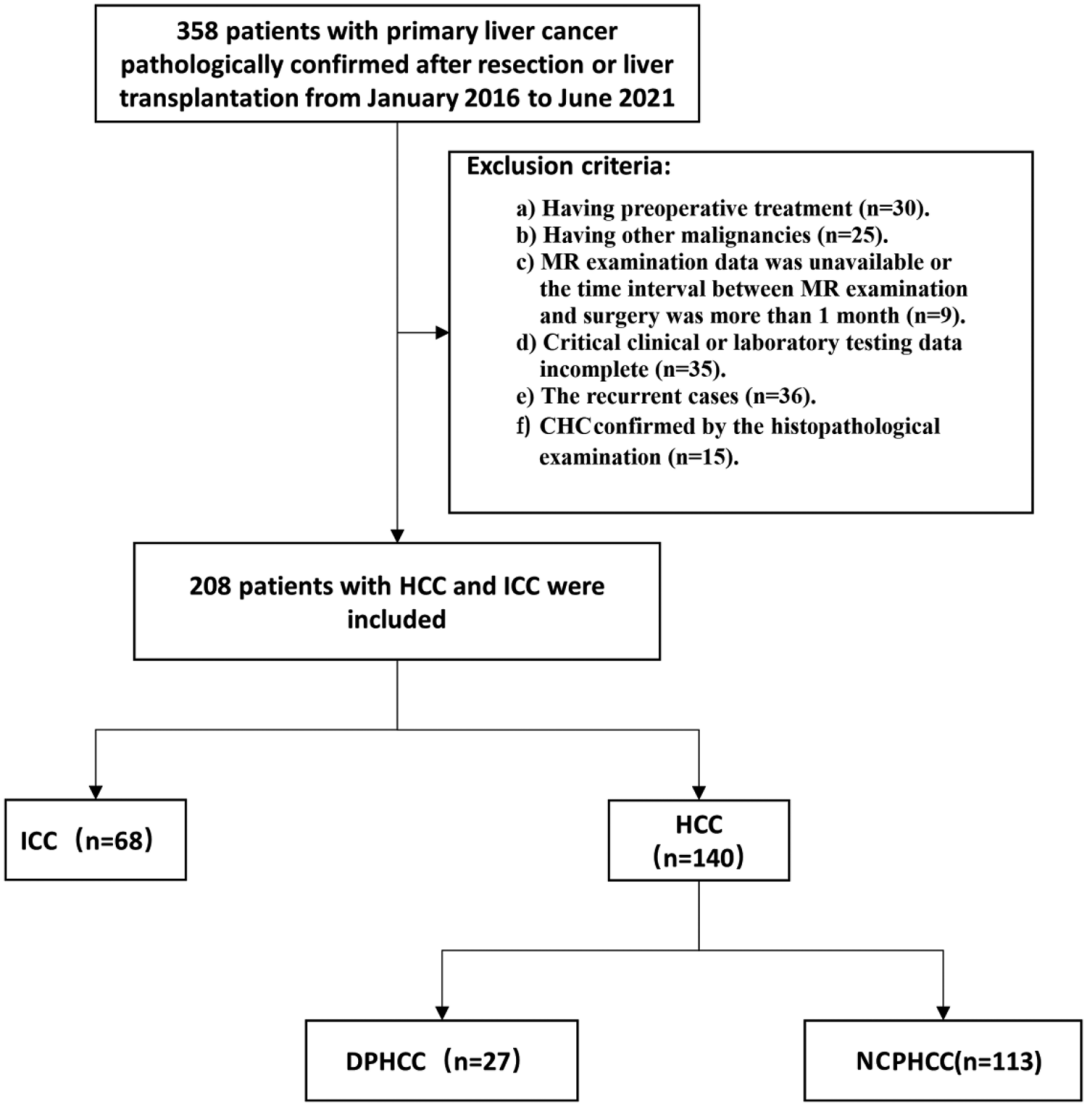
The study inclusion flow diagram

The exclusion criteria were as follows: (1) Previous treatment, such as chemoembolization, radiofrequency ablation or preoperative chemotherapy and radiotherapy (n = 30). (2) There was a history of other malignancies (n = 25). (3) There were no MR examination data available, or the time interval between MR examination and surgery was more than 1 month (n = 9). (4) The critical clinical or laboratory testing data were incomplete (n = 35). (5) The case was a recurrent case (n = 36). (6) CHC was confirmed by histopathological examination (n = 15) (Fig. 1).

### Immunohistochemistry

Histopathologic sections showed typical hepatocellular carcinoma. Immunohistochemical staining was performed on hepatocyte markers (e.g., Hep Par 1, GPC-3) and cholangiocyte markers (e.g., CK19, CK7). DPHCC is defined as the comoderate or strong expression of hepatocyte markers and cholangiocyte markers in more than 15% of the tumour cells.

### MRI Examinations

All MR abdominal examinations were performed on a 3.0 T system (MAGNETOM Trio Tim System Siemens Medical Solutions, Erlangen, Germany) or a 1.5 T system (SIGNA HDxt, GE Healthcare, Milwaukee, Wisconsin, USA or MAGNETOM Avanto System Siemens Medical Solutions, Erlangen, Germany). For dynamic-enhanced imaging, Gd-DTPA or gadoxetic acid disodium (Bayer Schering Pharma, Berlin, Germany, 0.1 mmol/kg) was injected intravenously, followed by a maximum dose of 20 ml saline flush at a rate of 2 ml/s. The hepatic arterial, portal, and delayed phase images were obtained at 25–30 s, 65–75 s, and 130–150 s, respectively, after contrast medium injection. Diffusion-weighted images with b values of 0 and 800 s/mm2 were acquired simultaneously by using respiratory-triggered single-shot echo-planar imaging before injection of the contrast agent. Apparent diffusion coefficient (ADC) maps were generated from a monoexponential function with b values of 0 and 800 s/mm2. Details of the sequence parameters are described in Table 1.

**Table 1:** MRI protocol and parameter of 1.5 T vs 3.0 T MR system

	TSE T2WI	EPI DWI	in/out phase T1WI	LAVA or VIBE T1WI
TR(ms)	4000 vs 3000	7200 vs 6300	6.1 vs 170	4.2 vs 3.67
TE(ms)	90 vs 84	71.4 vs 54	2.1/4.2 vs 1.3/2.5	2vs 1.34
NEX	3 vs 1	1 vs 1	1 vs 1	1 vs 1
Matrix	320 × 192	128 × 128	256 × 60	320 × 192
FOV(mm2)	380 × 380 vs 380 × 380	380 × 380 vs 380 × 380	380 × 400 vs 380 × 380	380 × 400 vs 380 × 380
Inversion angle	/ vs 111	/	12 vs 70	15 vs 12
Slice thickness(mm)	6 vs 5	6 vs 5	5 vs 5	5 vs 3
Slice gap(mm)	2 vs 1	2 vs 1	0 vs 1.2	0 vs 0.6

*T2WI* T2 Weighted Image, *T1WI* T1 Weighted Image, *DWI* Diffusion Weighted Imaging, *TR* Time of Repetition, *TE* Time of Echo, *NEX* Number of Excitations, *FOV* Field of View

### Image Analysis

Two board-certified radiologists with 9 or 10 years of experience in interpreting liver MR images reviewed all images and measured the quantitative parameters retrospectively and independently by a double-blind method according to the agreed uniform level and area of interest selection method before measurement. Quantitative parameters were averaged over the two-person measurements, and a consensus evaluation was performed when there was disagreement between the readers. The observers were blinded to the clinical data and previous imaging reports.

**Fig. 2:**
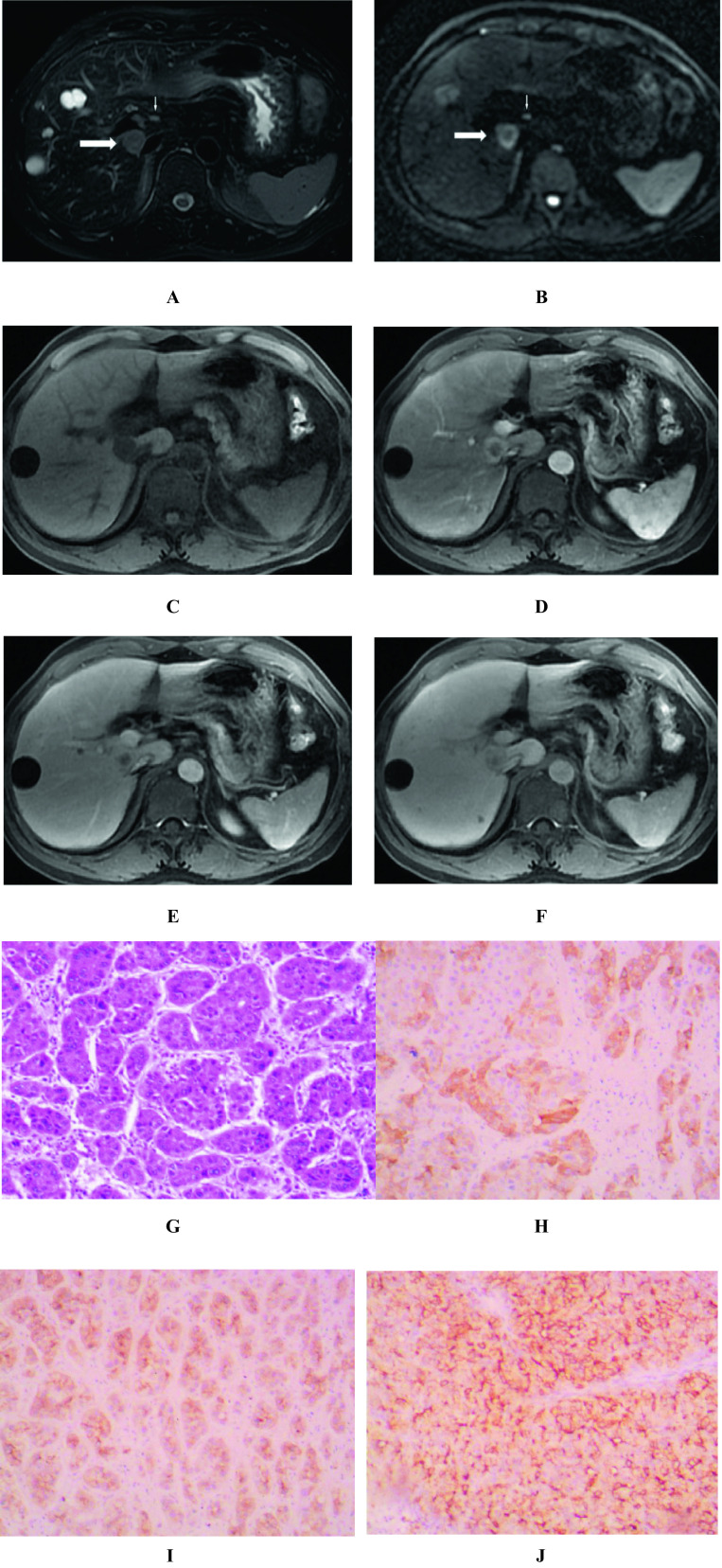
DPHCC (thick arrow) with hepatic hilar lymph node metastasis (thin arrow) and multiple liver cysts in a 46-year-old male with AFP 1261ug/L and positive hepatitis B-virus. T2-weighted image with fat suppression showed a moderately high signal mass with slightly higher signal margins than in the parenchyma of liver and lesion (**A**). Diffusion-weighted image (b = 800) showed a target sign, a high signal ring in the periphery of the mass and a slightly higher signal in the center (**B**). Tumor appears as low signal on T1-weighted image before the enhancement (**C**). In the enhanced scan, the tumor center showed no significant enhancement, displaying as persistent low signal. While the tumor periphery showed persistent enhancement, displaying as moderate enhancement in arterial phase (**D**), mild decreased enhancement in portal vein phase (**E**) and delayed phase (**F**). Photomicrograph (hematoxylin–eosin stain) showed a moderately to poorly differentiated HCC (**G**). Immunohistochemically, the tumor cells were positive for the cholangiocytic markers CK7 (**H**), CK19 (**I**), and hepatocyte marker GPC-3 (**J**)

**Fig. 3:**
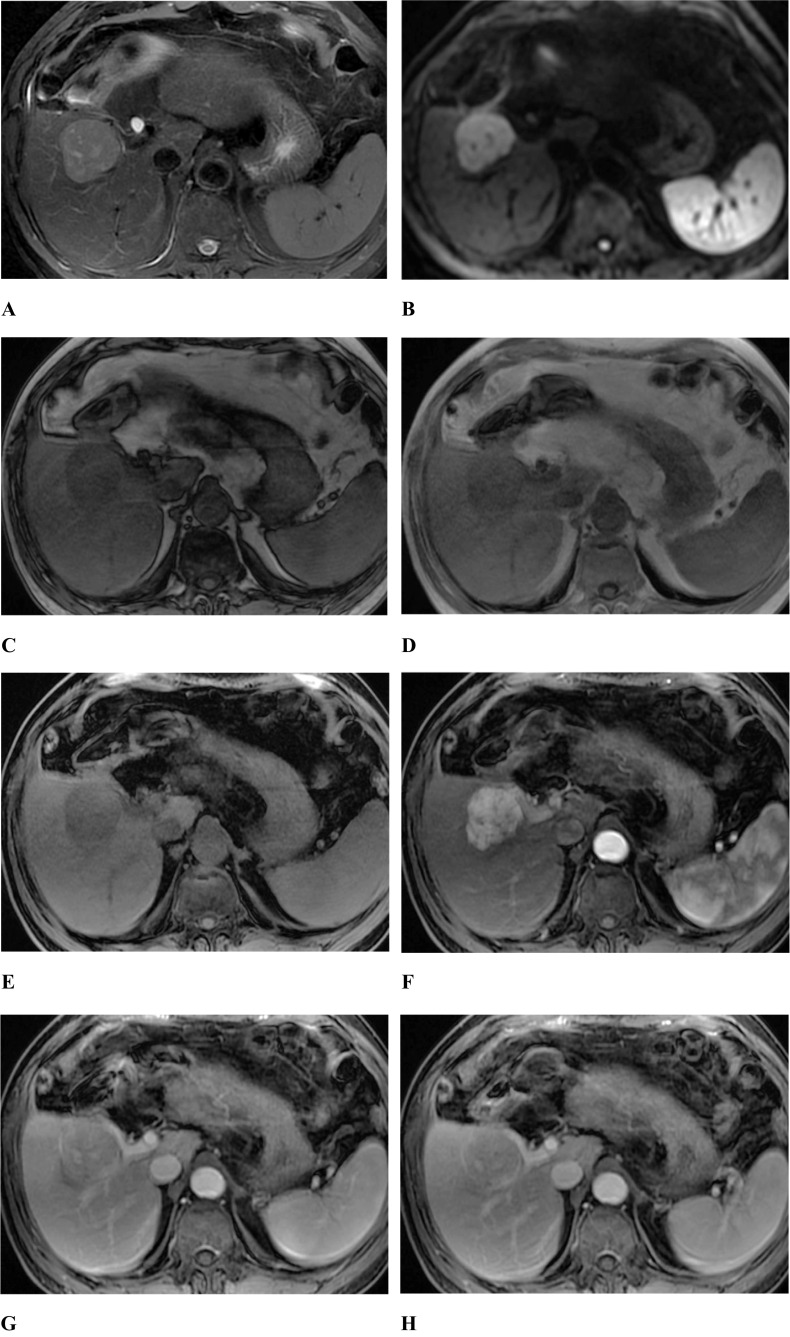
A 68-year-old male with NCPHCC. T2-weighted image with fat suppression showed a mass with slightly higher signal than in the hepatic parenchyma (**A**). Diffusion-weighted image (b = 800) showed a high signal mass (**B**). Tumor appeared as lower signal on out-phase image than on the in-phase image indicating steatosis, appeared as low signal on T1-weighted image with fat suppression before the enhancement (**C**). In the enhanced scan, the tumor showed fast-in and fast-out type, showing significantly enhancement in arterial phase (**D**), evidently decreased enhancement in portal vein phase (**E**) and false capsule with delayed enhancement in delayed phase (**F**)

**Fig. 4:**
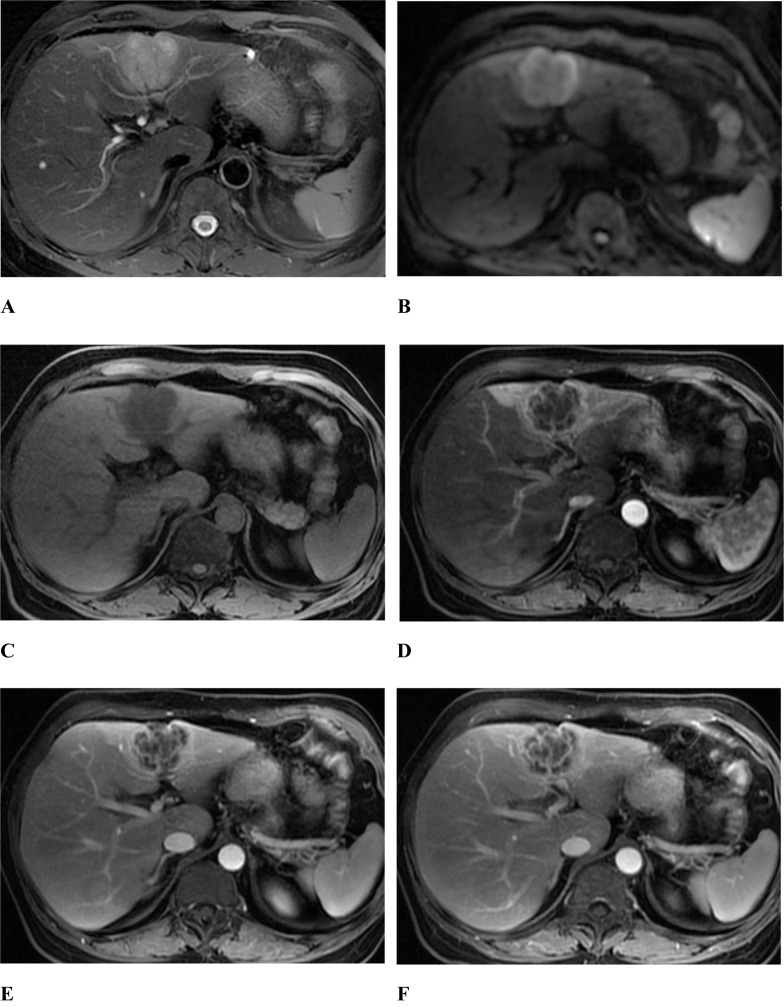
A 55-year-old female with intrahepatic cholangiocarcinoma. The tumor appeared as inhomogeneous hyperintense on T2 weighted image with fat suppression (**A**), target sign showing as a high signal ring periphery and slightly higher signal center on diffusion-weighted image (b = 800) (**B**). The lesion showed hypointense on precontrast T1 weighted image (**C**), peripheral irregular ring-like enhancement in the arterial phase (**D**), progressively honeycomb enhancement in the central part accompanied by a depressed hepatic capsule in the portal phase (**E**) and delayed phase (**F**)

The evaluated imaging parameters were as follows: (1) Number of lesions: in preoperative MR images, two or more tumour masses were considered multiple, but one lesion with a peripheral satellite focus (maximum diameter less than 1 cm) and at the same segment was considered single. (2) Tumor length: defined as the maximum diameter measured on the maximum cross section of the tumour on T2WI. (3) Tumour margin was categorized as smooth or not according to the budding part of the tumour margin on T2WI cross-section (4) The enhancement pattern was divided into three types: fast-in and fast-out types appeared as enhancement in the arterial phase and washout in the portal and delayed phases. The persistent type appeared as enhancement in the arterial phase and similar or slightly decreased enhancement during the portal and delayed periods. No or slight type appeared as no or slight enhancement in the arterial, portal and delayed periods. (5) Arterial rim enhancement: defined as the appearance of regular or irregular ring-like enhancement with central hypointense areas in the arterial phase. (6) Internal progressive small nodular enhancement: defined as, in the portal vein and in the delay phase, a small tuberosity-like enhancement in the unenhanced areas during the arterial phase. (7) Tumour capsule manifests as a low signal rim around the tumour in the arterial stage images but is enhanced in the portal stage and the delayed stage, and its length reaches more than 50% of the tumour circumference. (8) Haemorrhage: manifested as a high-signal area on T1WI with fat suppression (FS) or the typical changing haematoma signal over time on both T1WI and T2WI. (9) Necrosis or cystic degeneration: defined as a bright signal area on T2WI and no enhancement on T1WI FS sequences acquired in different phases after contrast medium administration. (10) Relatively high signal ring on T2WI: defined as a relatively high signal ring relative to the tumour parenchyma on T2WI. (11) Intratumor fat: tumour has an area with affirmatively decreased signal on T1WI with FS compared to without FS or on T1 out-phase image compared to T1 in-phase image. (12) Target sign on diffusion weighted imaging: defined as a ring-shaped hyperintense with central hypointense area on DWI (Figs. 2, 3 and 4). (13) Signal ratio on T2WI of the tumour to the erector muscle of the spine: defined as the signal ratio of the tumour region of interest (ROI) to the spinal erectus on the same level of T2WI, avoiding vascular, necrotic and cystic areas from being included in the ROI.

### Statistical Analysis

All statistical analyses were performed with the commercially available statistical software IBM SPSS software version 25.0. Statistical significance was set at p < 0.05. Continuous variables were compared using Student’s t test or the Mann‒Whitney U test. Categorical variables were compared using Fisher’s or continuity-corrected chi-square tests. First, the clinicopathological, laboratory and MRI data with P values < 0.05 were analysed by univariable logistic regression analysis to select variables that could help distinguish DPHCC from non-HCC or ICC. Next, variables with P < 0.05 in the univariate analysis were included in the multivariate logistic regression analysis to identify significant independent risk factors, and the odds ratio (OR) and 95% confidence interval (CI) were recorded. All variables were selected at once for multivariate analysis, and the variables with the largest p values and without significance were excluded each time until all variables reached the significance level.

## Results

### Demographic, Clinicopathological and Laboratory Characteristics of DPHCC Patients

Among 358 enrolled study participants, 150 were excluded because 1 or more exclusion criteria were met. The study inclusion flow diagram is presented in Fig. 1. Eventually, 27 patients (male to female ratio, 12:15) were included in the DPHCC group, 104 patients (male to female ratio, 104:9) were included in the NCPHCC group, and 68 patients (male to female ratio, 31:37) were included in the ICC group (Table 2).

**Table 2:** Differences in clinical laboratory characteristics between DPHCC, NCPHCC and ICC groups

Variables	DPHCC(a) (n = 27)	NCPHCC(b) (n = 113)	ICC(c) (n = 68)	P value (a *vs* b)	P value (a *vs* c)
Sex				0.000	0.920
Male	12(44.4%)	104(92.0%)	31(45.6%)		
Female	15(55.6%)	9(8.0%)	37(54.4%)		
Age(years)	54.78 ± 10.68	58.17 ± 10.31	59.57 ± 10.72	0.088	0.052
AFP > 20ug/L	18(66.7%)	60(53.1%)	4(5.9%)	0.202	0.000
CA199 > 37U/mL	3(11.1%)	11(9.7%)	46(67.6%)	1.000	0.000
CEA > 5 ug/L	5(18.5%)	11(9.7%)	25(36.8%)	0.341	0.084
CA125 > 35U/mL	4(14.8%)	19(16.8%)	38(55.9%)	1.000	0.000
HBV infection				1.000	0.000
Yes	26(96.3%)	108(95.6%)	10(14.7%)		
No	1(3.7%)	5(4.4%)	58(85.3%)		
hepatocirrhosis				0.297	0.000
Yes	19(70.4%)	90(79.6%)	9(13.2%)		
No	8(29.6%)	23(20.4%)	59(86.8%)		
MVI				0.601	0.020
Yes	5(18.5%)	14(12.4%)	30(44.1%)		
No	22(81.5%)	99(87.6%)	38(55.9%)		
PVTT				0.214	0.064
Yes	4(14.8%)	5(4.4%)	23(33.8%)		
No	23(85.2%)	108(95.6%)	45(66.2%)		
Lymphatic metastasis				0.364	0.000
Yes	4(14.8%)	8(7.1%)	40(58.8%)		
No	23(85.2%)	105(92.9%)	28(41.2%)		
Distant metastasis				0.214	0.064
Yes	4(14.8%)	5(4.4%)	23(33.8%)		
No	23(85.2%)	108(95.6%)	45(66.2%)		

There was a significant difference in the gender ratio between the DPHCC and NCPHCC groups (P < 0.001), while there was no significant difference in age, tumour marker levels, prevalence of HBV (hepatitis B virus) infection and incidence of hepatocirrhosis, microvascular invasion (MVI), portal vein tumour thrombus (PVTT), lymphatic and distant metastasis between the two groups (P > 0.05).

For the incidence of AFP > 20 µg/l, it was observed in 18/27 (66.7%) lesions in the DPHCC group and 4/68 (5.9%) in the ICC group (P < 0.001). In the DPHCC group *vs.* ICC group, the incidence of CA199 > 37 U/ml, CA125 > 35 U/ml, CEA > 5 µg/l, and PVTT was 11.1% *vs.* 55.9% (P < 0.001), 14.8% *vs.* 5.9% (P < 0.001), 18.5% *vs.* 36.8% (P > 0.05), and 14.8% *vs.* 33.8% (P > 0.05) of lesions, respectively. In addition, the results showed that the prevalence of HBV infection and incidence of hepatocirrhosis in the DPHCC *vs.* ICC group were 96.3% *vs.* 14.7% (P < 0.001) and 70.4% *vs.* 13.2% (P < 0.001), respectively. At pathologic analysis, there was a significant difference in MVI and lymphatic metastasis (P = 0.020 and P < 0.001, respectively), while there was no significant difference in distant metastasis (P = 0.064) between the two groups.

### MRI Findings of DPHCC

When comparing MRI results (Table 3), nonsmooth tumour margin, T2WI higher signal intensity, DWI target sign, persistent enhanced type, intratumoral progressive small nodular enhancement, and arterial phase rim enhancement were more observed in the DPHCC group than in the NCPHCC group (70.4% *vs.* 18.6%, 22.2% *vs.* 8.8%, 77.8% *vs.* 6.2%*,* 70.4% *vs.* 5.3%, 74.1% *vs.* 4.4%, 74.1% *vs*8.0%, P < 0.001, respectively) (Fig. 2). Tumour capsule, intratumoral fat, and the fast-in and fast-out enhanced type were more common in the NCPHCC group than in the DPHCC group (72.6% *vs.* 29.6%, 55.8% *vs.* 7.4%, 94.7% *vs.* 29.6% P < 0.001, respectively) (Fig. 3), while there were no statistically significant differences in the number of lesions, tumour length, the incidence of intratumoral haemorrhage, necrosis or cystic degeneration, or slightly higher signal ring on T2WI between the DPHCC group and NCPHCC group (P > 0.05). To extract the factors associated with DPHCC, further analyses by univariate and multivariate were performed (Table 4). Nonsmooth tumour margin, high signal ratio of tumour to erector spinae muscle on T2WI, target sign on DWI, absence of tumour capsule, persistent enhancement, intratumoral progressive small nodular enhancement, and arterial phase rim enhancement were significant risk factors for DPHCC in the univariable linear regression analysis. To extract the factors associated with DPHCCs, the factors with P < 0.05 were included in the multivariate analysis. The absence of tumour capsule (OR = 9.777, P = 0.046), persistent enhancement (OR = 46.941, P = 0.006), and target sign in DWI (OR = 30.566, P = 0.021) were independent risk factors associated with DPHCC in the multiple regression analysis.

**Table 3:** Differences in MRI performance between the DPHCC, NCPHCC and ICC groups

Variables	DPHCC(a) (n = 27)	NDPHCC(b) (n = 113)	ICC (c) (n = 68)	P value (a *vs* b)	P value (a *vs* c)
Tumor				0.570	0.071
Single	19(70.4%)	73(64.6%)	34(50.0%)		
multiple	8(29.6%)	40(35.4%)	34(50.0%)		
The larger tumor length (cm)	4.62 ± 3.02	4.76 ± 3.49	6.69 ± 3.54	0.914	0.006
Tumor margin				0.000	0.000
smooth	8(29.6%)	92(81.4%)	1(1.5%)		
non-smooth	19(70.4%)	21(18.6%)	67(98.5%)		
Tumor capsule				0.000	0.073
present	8(29.6%)	82(72.6%)	8(11.8%)		
absent	19(70.4%)	31(27.4%)	60(88.2%)		
Hemorrhage				0.243	1.000
present	1(3.7%)	16(14.2%)	3(4.4%)		
absent	26(96.3%)	97(85.8%)	65(95.6%)		
Necrosis or cystic degeneration				0.787	0.311
present	12(44.4%)	47(41.6%)	21(30.9%)		
absent	15(55.6%)	66(58.4%)	47(69.1%)		
Intratumor fat				0.000	0.166
present	2 (7.4%)	63(55.8%)	15(22.1%)		
absent	25(92.6%)	50(44.2%)	53(77.9%)		
Contrast enhancement pattern				0.000	0.001
Fast in and fast out	8(29.6%)	107(94.7%)	2(2.9%)		
Persistent	19(70.4%)	6(5.3%)	66(97.1%)		
Intratumor progressive small nodular enhancement				0.000	0.016
present	20(74.1%)	5(4.4%)	64(94.1%)		
absent	7(25.9%)	108(95.6%)	4(5.9%)		
Arterial phase rim enhancement				0.000	0.024
present	20(74.1%)	9(8.0%)	33(48.5%)		
absent	7(25.9%)	104(92.0%)	35(51.5%)		
Slightly higher signal ring on T2WI				0.104	0.092
present	6(22.2%)	10(8.8%)	5(7.4%)		
absent	21(77.8%)	103(91.2%)	63(92.6%)		
Tumor to erector spinae muscle signal ratio on T2WI	3.54 ± 1.76	2.22 ± 1.20	4.03 ± 2.45	0.000	0.070
Target sign on DWI				0.000	0.329
present	21(77.8%)	7(6.2%)	46(67.6%)		
absent	6(22.2%)	106(93.8%)	22(32.4%)

**Table 4:** Value of univariate and multivariate analysis in the diagnosis of DPHCC and NCPHCC

Variables	Univariate Analysis		Multivariate Analysis	
	P value	OR (95% CI)	P value	OR (95% CI)
Tumor margin	0.000	10.405(4.014, 26.968)		
Tumor capsule	0.000	6.282(2.494, 15.822)	0.046	9.777(1.037, 92.218)
Intratumor fat	0.202	2.688(0.588, 12.280)		
Contrast enhancement pattern	0.000	42.354(13.204, 135.859)	0.006	46.941(3.005, 733.275)
Intratumor progressive small nodular enhancement	0.000	61.724(17.808, 213.873)		
Arterial phase rim enhancement	0.000	33.016(11.017, 98.943)		
Tumor to erector spinae muscle signal ratio on T2WI	0.000	0.547(0.404, 0.740)		
Target sign on DWI	0.000	53.000(16.177, 173.636)	0.021	30.566(1.678, 553.970)

In the comparison of the imaging findings between the DPHCC group and the ICC group (Fig. 4), there were no significant differences in the number of lesions, absence of tumour capsule, intratumoral haemorrhage, necrosis or cystic degeneration, intratumoral fat, slightly higher signal ring on T2WI, the signal ratio of tumour to erector spinae muscle on T2WI, and target sign in DWI (P > 0.05). There were significant differences between the DPHCC group and the ICC group in nonsmooth tumour margin (70.4% *vs.* 98.5%, P = 0.006), persistent enhanced type (70.4% *vs.* 97.1%, P = 0.006), arterial phase rim enhancement (74.1% *vs.* 48.5%, P = 0.044), and intratumoral progressive small nodular enhancement (74.1% *vs.* 94.1%, P = 0.011). To extract the factors associated with DPHCCs, imaging findings and clinical laboratory data with P < 0.05 were included in the multivariate analysis. AFP > 20 µg/L (OR = 67.097, P = 0.036) and prevalence of HBV infection (OR = 153.633, P = 0.020) were independent risk factors correlated with the DPHCC (Table 5).

**Table 5:** Value of univariate and multivariate analysis in the diagnosis of diagnosis of DPHCC and ICC

Variables	Univariate Analysis		Multivariate Analysis	
	P value	OR (95% CI)	P value	OR (95% CI)
AFP>20ug/L	0.000	23.000(6.282, 84.203)	0.036	67.097(1.307, 3444.311)
CEA>5ug/L	0.043	3.186(1.038, 9.782)		
CA125>35U/mL	0.001	7.318(2.205, 24.289)		
CA199>37U/mL	0.000	17(4.455, 64.876)		
Hepatitis	0.000	190.667(21.731, 1672.936)	0.020	153.633(2.176, 10848.946)
hepatocirrhosis	0.000	17.417(5.312, 57.101)		
Tumor margin	0.006	20.632(2.415, 176.285)		
Contrast enhancement pattern	0.006	10.105(1.964, 51.992)		
Intratumor progressive small nodular enhancement	0.011	0.179(0.047, 0.673)		
Arterial phase rim enhancement	0.044	2.857(1.026, 7.954)		

## Discussion

Hepatocytes, bile duct epithelial cells and hepatoma carcinoma cells all contain cytoskeleton intermediate filaments. Different types of cancer cells have different characteristic combinations of cytokeratin (CK), in which hepatocytes and common hepatocellular carcinoma usually express CK8 and CK18, while cholangiocytes and cholangiocarcinoma usually express CK7 and CK19. Although both CK7 and CK19 are expressed in cholangiocytes, CK7 is also recognized as a marker of intermediate hepatocytes, and CK19 acts as a marker of ICC and biliary differentiation. In recent years, some articles reported that CK7 and CK19 have also been shown to be expressed in partial HCC, and this type of HCC was named DPHCC. DPHCC is a newly defined subtype of HCC in recent years, and was proposed as a routine diagnosis of pathology in the guidelines for the standardized pathological diagnosis of primary hepatocellular carcinoma (2015 edition) [8], which is mainly characterized by the expression of both HCC and ICC biomarkers within the same cancer cell. With the accumulation of knowledge on the molecular characteristics and prognostic significance of DPHCC, this particular HCC subtype has received increasing clinical attention, and some studies have confirmed its higher malignancy and more aggressive nature due to its dual biological characteristics of HCC and ICC [6, 7, 9, 10]. Although it is difficult to make a definite diagnosis of DPHCC before operation, it is helpful to select and improve the treatment if there is any doubt or hint. Therefore, how to detect or prompt DPHCC early by noninvasive methods has become the focus of clinical attention.

Our findings suggest that compared with NCPHCC, absence of tumour capsule, persistent enhancement, and target sign in DWI were independent risk predictors of DPHCC. In addition, compared with ICC, AFP > 20 µg/L and prevalence of HBV infection were significant independent factors for predicting DPHCC. DPHCC may have some imaging findings of ICC and some clinical laboratory findings of NCPHCC, and integrating the imaging characteristics of ICC and the clinical laboratory data of HCC may be valuable for predicting DPHCC in clinical practice.

Capsule formation is one of the major imaging features of progressive HCC [11], and its formation mechanism is not fully understood. The accepted mechanism is that the expansion growth of tumour cells compresses adjacent liver tissue, leading to fibrous desmoplasia. At the same time, tumour cells and their stromal cells produce a large number of cytokines that promote angiogenesis, and angiogenesis in fibrotic connective tissue also promotes the formation of the tumour capsule [12, 13]. Previous studies have shown that HCC with an intact tumour capsule has a better prognosis after resection or radiofrequency ablation and that a tumour capsule is a favourable prognostic factor for HCC [14]. It has been shown that HCC without a capsule is more malignant and invasive [15]. In this study, we found that the absence of a tumour capsule was an independent risk factor for DPHCC and that patients without a tumour capsule were more common in the DPHCC group than in the NCPHCC group. Furthermore, the incidence of nonsmooth tumour margins was also high in the DPHCC group. In the DPHCC *vs.* NCPHCC groups, the incidence of MVI, PVTT and lymphatic metastasis was 18.5% *vs.* 12.4%, 18.5% *vs.* 4.4% and 14.8% *vs.* 7.1%, respectively, but with no significant difference, which may be related to the small case sample included in this study. These results strongly suggest that DPHCC is more aggressive. Preoperative targeting, somatostatin and its analogues may contribute to improving the efficacy and prognosis of DPHCC.

In this study, we found that most DPHCC exhibits a progressive enhancement patterns relative to NCPHCC, which is also supported by previous studies [10]. While the persistent enhancement pattern is a typical image feature of the ICC [16, 17], our results showed no statistically significant difference in the enhancement pattern between the DPHCC and ICC groups. Expression of CK19 by partial tumour cells is an important feature of DPHCC, and CK19 is a progenitor cell marker that is thought to reflect the cholangiocyte differentiation of hepatoma cells and can lead to the production of interstitial connective tissue within the tumour [18]. This cholangiocyte differentiation component may contribute to the prolonged contrast agent retention time in DPHCC, exhibiting the findings seen in ICC imaging. Furthermore, we found that the target-like sign on DWI was an independent significant factor of DPHCC, and that the incidence of arterial phase enhancement at the tumour margin was 74.1% *vs.* 48.5% in the DPHCC *vs.* ICC groups, respectively. Previous studies showed that the target-like signs on DWI and the marginal arterial phase enhancement of the tumours were identified as the independent variable factors in the diagnosis of small ICC [19, 20]. CK19 could be a potential marker of the therapeutic benefit of reggrafenib, and the CK19 phenotype may have a crucial role in predicting poor prognosis and preserving the malignant properties of HCC [21]. We speculate that DPHCC with some imaging features similar to ICC may be related to CK19 expression in tumours, so that the imaging findings may also be useful for the evaluation of CK19 expression and the prognostic evaluation.

In addition, we found that AFP > 20 µg/L and the prevalence of HBV infection were independent risk factors for predicting DPHCCs compared to ICCs, while there were no significant differences between the DPHCC group and the NCPHCC group, which is consistent with previous findings [6]. This may be related to the high prevalence of HBV infection in DPHCC patients, as in HCC patients. On the other hand, it has been suggested that because DPHCC cells can exhibit bipotential expression similar to liver progenitor cells, serum AFP and CA199 proteins may be elevated simultaneously, suggesting that DPHCC may originate from liver progenitor cells and thus have dual immunohistological characteristics of HCC or ICC [6]. In this study, no significant differences were found between the DPHCC and NCPHCC groups in CA199 > 37 U/mL, which may be related to the small number of cases in this study. Of the 68 ICC patients, 4 (5.9%) had AFP > 20 µg/L. A review of the patients’ histories found that these 4 patients had hepatitis or cirrhosis, which may be responsible for the elevated AFP.

In summary, we can obtain preoperative information indicating DPHCC through multimodal MRI imaging performance (including no tumour capsule, persistent enhancement pattern, and target sign on DWI) and clinical laboratory tests (including serum AFP > 20 µg/L and HBV infection prevalence). Therefore, a comprehensive evaluation of MR imaging findings and clinical laboratory features, rather than relying solely on imaging or clinical laboratory features, can provide valuable information for the diagnosis and prognosis assessment of different HCC types. However, in clinical analysis, subjective or objective factors can easily interfere, so it is difficult to properly integrate the above factors in diagnosis. In the future, the construction of a large multicentre database and the strengthening of machine learning and supervision will help to build an accurate preoperative diagnosis and evaluation model for DPHCC.

Our study has several limitations. First, the inclusion of primary cancer histopathologically confirmed through surgery or liver transplantation might have introduced a selection bias, and HBV infection prevalence could be due to a general HBV infection prevalence in the studied population. Furthermore, the main risks of hepatitis C virus (HCV) infection and alcoholic HCC were not included in the statistical analysis. Second, two different MR systems were used in this study, and the inconsistency of the imaging parameters may have affected the consistency of the image features. The sample size of DPHCC is small, and there may be some differences in the degree of simultaneous expression of bile duct cell markers and hepatocyte markers in DPHCC cells, which may affect the statistical results of some parameters. Third, because the sample was too small, our study did not investigate the imaging characteristics of CHC, the effect of CK7 expression and the imaging findings of primary liver cancer in the contrast-enhancing hepatobiliary-specific stage, as in other state-of-the-art MR multiparametric imaging studies. Finally, because the CHC samples collected were too small or the image data were incomplete, this study did not include CHC in the controlled study and did not compare the influence of CK7 expression, liver-biliary specific enhancement and other MR image parameters. Therefore, our study results still need multicentre prospective validation with large samples to provide an important noninvasive predictor of DPHCC and may contribute to treatment regimen selection and prognostic evaluation for different types of liver cancer patients. Therefore, the results of our study need to be prospectively verified by multicentre and large-sample studies in the future to provide an important noninvasive diagnostic model and predictive index for DPHCC to achieve accurate treatment and prognosis assessment for patients with different types of liver cancer.

Additional notes on this article: Artificial intelligence can help objectively measure and evaluate tumour imaging information and heterogeneity, providing objective information for personalized diagnosis and treatment. Recently, deep learning techniques have even reached expert-level performance in a variety of medical image analysis tasks [22–24]. However, image-based AI has not yet been successfully applied to the precise diagnosis and treatment of liver tumours. Although the two radiologists in this study had 9 or 10 years of experience in liver MR image interpretation, their measurements of DPHCC image parameters were consistent only after they first discussed the unified method. When they analysed the imaging, clinical, and laboratory features of the patients, they agreed that all DPHCC cases were preoperatively suggestive. The results of this study suggest that the construction of an AI comprehensive evaluation model based on imaging, clinical and laboratory characteristic parameters will be conducive to integrating all key factors, reducing the interference of subjective or one-sided factors, achieving accurate evaluation of HCC subtypes, and helping precision medicine.

## Data Availability

The data used to support the findings of this study are available from the corresponding author upon request.
